# Her Heart's Mistake

**Published:** 1889-06-29

**Authors:** E. D. Cumming

**Affiliations:** Author of "An Ordeal by Silence"


### HER HEART'S MISTAKE.

By E. D. CUMMING, Author of "An Ordeal by Silence."

*(Continued from page 189.)*

### CHAPTER XI.—A VISIT, AND A LITTLE DINNER.

THE drawing-room of the corner bungalow was full of visitors. A number of Kate Harrison's friends had selected the same day to come round and have a quiet chat with her, and fully half-a-dozen ladies were present, including Mrs. Macdonald, the Collector's wife, who had just returned from her sojourn in the hills, and who had made it her first duty to call and congratulate her dear Katie upon her engagement.

"Macdonald and I were just as pleased as pleased could be," said the lady, enfolding Kate in a comprehensive embrace, and inflicting a succession of resounding kisses upon her features at random, "and Macdonald (who only put in a fortnight with me at Mussoorie, and is off on his travels in the district again) told me to say that *he* congratulates Captain Bairdsley."

And having delivered her message, Mrs. Macdonald sat down on the arm of a long chair, loosened her bonnet-strings, and called loudly upon the invisible punkah-wallah to exert himself. For the day was very hot, and she was unbecomingly warm.

"There's no more weddings promised, I suppose!" she said, beaming round upon the assemblage with a fat smile. "They say that one wedding makes many. What about Captain Phelps, Gerty Walford?"

Mrs. Pringle hastily called the blushing Miss Walford to her side, and thus enabled her to retreat in fairly good order without replying to this painfully direct fire.

"I do not think that there are any more engagements announced," said Mrs. Strachan, speaking with laborious distinctness of articulation. "I hope you enjoyed yourself at Mussoorie, Mrs. Macdonald?"

"O it's fine place is Mussoorie, Anna Strachan; I was just as happy as happy could be up there, but I'm very pleased to be down here again, hot as it is," replied the Collector's wife, using a rolled-up handkerchief as a fan. "Katie, my dear, just stir up your punkah-wallah; he doesn't mind me."

Miss Harrison went out to the back verandah, "stirred up" the servant, as requested, and re-entered the drawing-room to see a figure, dressed in a black coat and waistcoat and tweed trousers, crossing the threshold, which she recognised in the dim light as Mr. Warren's. He hesitated slightly when he saw the gathering of ladies presided over by Mrs. Macdonald, and Kate came forward to greet him.

"How do you do, Mr. Warren?" she said. "Papa told us we might hope to see you before long."

Mr. Warren was clearly a retiring and diffident man. He was red when he came in, owing to the heat; but the dead silence which his appearance caused for a minute made him turn a rich purple.

"Mrs. Macdonald, may I introduce Mr. Warren, who has just arrived from home," said Kate, presenting him to the head of Meerut society.

"How do you do, Mr. Warren?" said that lady, shaking hands with great warmth. "You look hot."

"It is very hot," said the gentleman, feeling by no means cooler on receipt of the information. He had been told that Mrs. Macdonald was the "burra memsahib," or "great lady" of the station, and he had been prepared to meet a stately personage of majestic bearing, before whom common people like himself should bow the knee. He was not good at making formal acquaintances, such as he anticipated Mrs. Macdonald would be, and it had been a real relief to him when he was informed at her bungalow ten minutes ago that she was out, and found that the object of his call was to be attained by simply writing his name in the visitor's book. The apparition, seated on the arm of the chair, startled him, and the reception she gave him was not what he had anticipated. Excessive hauteur and extreme familiarity are equally embarrassing under such circumstances, and Mr. Warren having admitted that it was hot, stood before her, nervously hugging his solah topee, absolutely at a loss to know what to say next.

"When you come to see me, Mr. Warren, you'll come at a more agreeable hour, and not trouble to put on your best clothes."

"I did myself the pleasure of calling at your bungalow to-day," said poor Mr. Warren, whose nervousness was not decreased by the faint suspicion of a giggle which came from Mrs. Strachan's direction; "and was sorry to find you were out."

"I hope they offered you a peg?" said Mrs. Macdonald, quickly. "I'm always telling that khansamah of mine to offer callers a peg when I'm out or not receivin'. They want it this weather."

"I was not in the least thirsty, thank you very much, Mrs. Macdonald," said Mr. Warren, who was beginning to brace himself up for what he saw was likely to be an ordeal. "It is most kind of you to think of such a thing."

"Did the khansamah offer it to you?" insisted Mrs. Macdonald.

"No, he did not; but"——

"That fellow's tullub is cut eight annas, and I won't forget it on pay-day," said the lady. "Why don't you sit down, Mr. Warren? The punkah's making your hair so untidy."

The unhappy gentleman sank into a low folding-chair, and assumed the attitude usually struck by men when they first take their seats in church, that he might blush into his solah topee unseen.

"Had you a good voyage out, Mr. Warren?" enquired Mrs. Macdonald, after a brief pause.

"Very fair until we got to Aden; it was fearfully rough afterwards all the way to Bombay." He emerged from cover to make the reply, but the next query sent him back to it, more embarrassed than ever.

"Were you sick?" asked Mrs. Macdonald, in a loud whisper, raising her eyebrows with a smile which was intended to invite confidence, but which served to attract the attention of the whole room.

At this moment, however, Kate earned his eternal gratitude by coming to rescue him from the clutches of this terrible female. She sat down at his side and set to work diligently to put him at his ease.

"Papa tells me that your father was a very dear friend of his in bygone days, Mr. Warren," she began, "and my aunt, Mrs. Brent, says she remembers you at Devonport. She would like to have seen you, but she is obliged to lie down during the heat of the day."

"I recollect both Dr. Harrison and his sister—she was Miss Harrison in those days. I was very glad to find that there were some old friends at Meerut when I decided to join Mr. Pringle here."

Kate held him in conversation for a few minutes and seized an opportunity to introduce him to Mrs. Strachan—Mrs. Pringle's acquaintance he had already made—and employed herself with Mrs. Macdonald, who was evincing signs of impatience to obtain possession of her victim again. Mrs. Strachan was not impressed with the new comer; he was awkward and silent, answering her in monosyllables, and never volunteering a remark.

"A perfect stick, my dear, a regular stick," she told Mrs. Markham, in answer to questions that evening at the club. "Mrs. Macdonald was there, and took him in hand in her usual fashion, and he was quite crushed. Hadn't a word to say; didn't even try to laugh it off. I was sorry for him at first, but when Kate introduced him to me and I found out what he was, I was very much inclined to let the burra mem have him to play with again. I laughed outright, he got so confused; I simply could not help it."

"Mrs. Macdonald is rather a trying ordeal sometimes," said Mrs. Markham, "and I can imagine what it must be for a shy man who meets her for the first time before a lot of women."

"But, my dear Mrs. Markham, no man of the world would have been utterly abashed as Mr. Warren was. I assure you, he looked as though he longed for the floor to open and swallow him up."

"He must be very shy."

"I don't see why he need have been shy with me," said Mrs. Strachan. "He sat beside me for fully ten minutes, and I don't believe he said a dozen words the whole time. One expects something better of a barrister. They generally have quite enough to say for themselves."

As a matter of fact, this very shyness of which Mrs. Strachan complained was in no one's way more than that of Richard Warren himself. He was a man of unusual ability, and had been called to the Bar at an earlier age than the majority of those who adopt law as a profession. He was a clear-headed vigorous-minded man, whose powers of concentration and grasp would have ensured success, not only complete but brilliant, if that unfortunate and unconquerable diffidence had not deprived him of fluent speech. He might have risen to the greatest heights of the profession, said his legal friends, if only he had been able to speak for five minutes without breaking down from sheer nervousness. He was a sound lawyer, they said, but would never take the position his talents entitled him to.

He had no private fortune, and the livelihood he earned in England did not satisfy him; so he cast about for an opening in the East, and Mr. Pringle's offer to share his practice with him, on advantageous terms, came at the proper time. He sold off his furniture, packed up his library and clothes, bade his only living relative farewell, and took passage for India, feeling certain that a man of his temperament would have a better chance of success in courts where the presiding judge and a few European officials replaced the crowded halls of justice, in which he could not accustom himself to argue freely at home. He was popular among men. Those who knew him well were wont to say that Dick Warren was one of the few people whose acquaintance repaid cultivation; for life was too short to spend much time in getting at deep men generally.

He had mixed but little in ladies' society, for the overweening modesty, which was his most patent characteristic, did not conduce to his popularity among women. He was well known by report as a clever man, and laughter-loving girls who took it for granted that he must of necessity be "amusing," that being synonymous in their vocabulary with cleverness, prepared disappointment for themselves when they sought to have him introduced. Dick Warren's talents were not of a superficial order, and did little to recommend him to the favourable notice of those who base their judgment more on manner than character. He was certainly very awkward in a drawing-room. He never knew what to do with his hat, and those big hands of his always seemed to be in his way. He was not ornamental, nor, in that sphere at all events, useful; so it came about that invitations to at homes and dances grew gradually fewer and farther between. His name disappeared mysteriously from ladies' "lists," and the owner from their ken. And Dick Warren smiled at it, put on his old coat and slippers, lit his pipe, buried himself in his book, and was happy. For several years prior to his departure for India he had given up most forms of gaiety, and sought congenial associates among men and women of his own cast of mind.

Small wonder, then, that Mrs. Macdonald's breezy affability made his lack of worldly ready-wittedness appear in the strongest light, and gave Meerut society the false keynote to his real character at the outset. Like every other new arrival, he paid the usual round of formal calls upon the residents, and though he was everywhere received with Anglo-Indian cordiality and kindness, the knowledge that his first interview with the Collector's wife was common property soon came home to him, and did nothing to enhance the very slight avidity with which he carried out the task.

"Have you seen Mrs. Macdonald yet?" his hostess would ask, in accents which betrayed that she knew he had, and wanted to hear what he thought of her.

"I met her at Dr. Harrison's the other day," he would reply, with a shudder at the recollection.

"She is a little peculiar in her manner, as you no doubt discovered, Mr. Warren," was the remark which, in slightly varying phraseology, invariably followed his admission. And when, with his natural caution, he declined to be "drawn" on the subject, he was consoled by the information that that was merely her way, and that she treated everyone alike. Then Dick Warren would say good-bye, climb into his gharry, and drive away to the next house, weighed down by the thought that he had initiated his appearance in Meerut by making a fool of himself, and that everybody knew it.

"Kate, my dear," said the Doctor, one morning, "you must fix an evening for Warren to come and dine with us, and ask a few people to meet him."

"It will be rather an undertaking, papa; he is fearfully heavy and silent."

"That can't be helped, child. I am anxious to show him attention on his father's account, and I am much mistaken, moreover, if that young man is so stupid as he is thought. Pringle has a very high opinion of him, and I like what little I've seen of him myself."

"He has never thought it necessary to come over to our rooms of an evening at the club," said Mrs. Brent resentfully. "I have never even spoken to the man yet. And, considering that we know his belongings, I think it shows a want of manner on his part."

"Well, well, Agnes, you will be able to judge him for yourself ere long. Suppose you make up a little party for Friday week, Kate."

Miss Harrison accepted the suggestion as a command, and when breakfast was over adjourned with her aunt to decide upon the people to be asked. It was not a difficult matter to make up the party, inasmuch as it was to be a somewhat formal affair, whose object was to introduce Mr. Warren to Meerut society. Mrs. Macdonald, the Strachans, the Tenterdales, the Pringles, and Major Brooks, the Brigade Major, completed the list; and Kate felt that if she could only arrange to keep the "burra mem-sahib" away from Mr. Warren she would be able to pull through the dinner somehow in spite of his terrible heaviness.

She was a good hostess, and when she found him committed to her care for the meal devoted herself gracefully to the task of entertaining him. She tried him upon light topics first, but found that his tastes were not of a frivolous order; he did not dance, could not play or sing, and had never been in the saddle since he was a boy. He intended to try and get some big game shooting if he could manage it, and had Kate been able to talk "shikar" with him she thought he would have come out a little, but she knew nothing of such matters and had to fall back upon her favourite books. Here she found common ground with him at last; he had read everything she had read, everything she could suggest, and a vast number of books she had never heard of. At the beginning of the meal she had done all the talking, but long before it was half over she was listening attentively to him; he forgot his shyness and did not notice that their conversation was hearkened to by all the guests at their end of the table. Kate had referred to her last favourite, "The Story of an African Farm," and he was discussing the marvellous truth of Lyndall's speech upon women's place in the world. His sympathies were all with her, and he dwelt upon the difficulties and temptations by which women, condemned by social custom to pretty idleness were surrounded, with force and point. How few succumbed to those temptations proved the greater strength of their moral nature as compared to man's. The far greater and better influence women might wield were their restraints relaxed. He spoke with a vigour and freedom which astonished her, and commanded Kate's respect. This man was not the dull clown he had appeared to be, far from it. There was mental power here, and a mind of no ordinary class; she drank in his words as he spoke, and promised herself many long talks with Mr. Warren in the future. He was a different stamp to the men she was used to meeting; he drew her out of herself, and brought out in speech what had hitherto been dormant thought. She felt new ideas taking birth in her mind, and so absorbed was she in the man's conversation that her aunt had to send a servant round to remind her that it was time for her to give the signal for the ladies to leave the table. She gave it unwillingly, and rose from her seat, with an opinion of Richard Warren which was very different to that she had held when she sat down.

*(To be continued.)*

